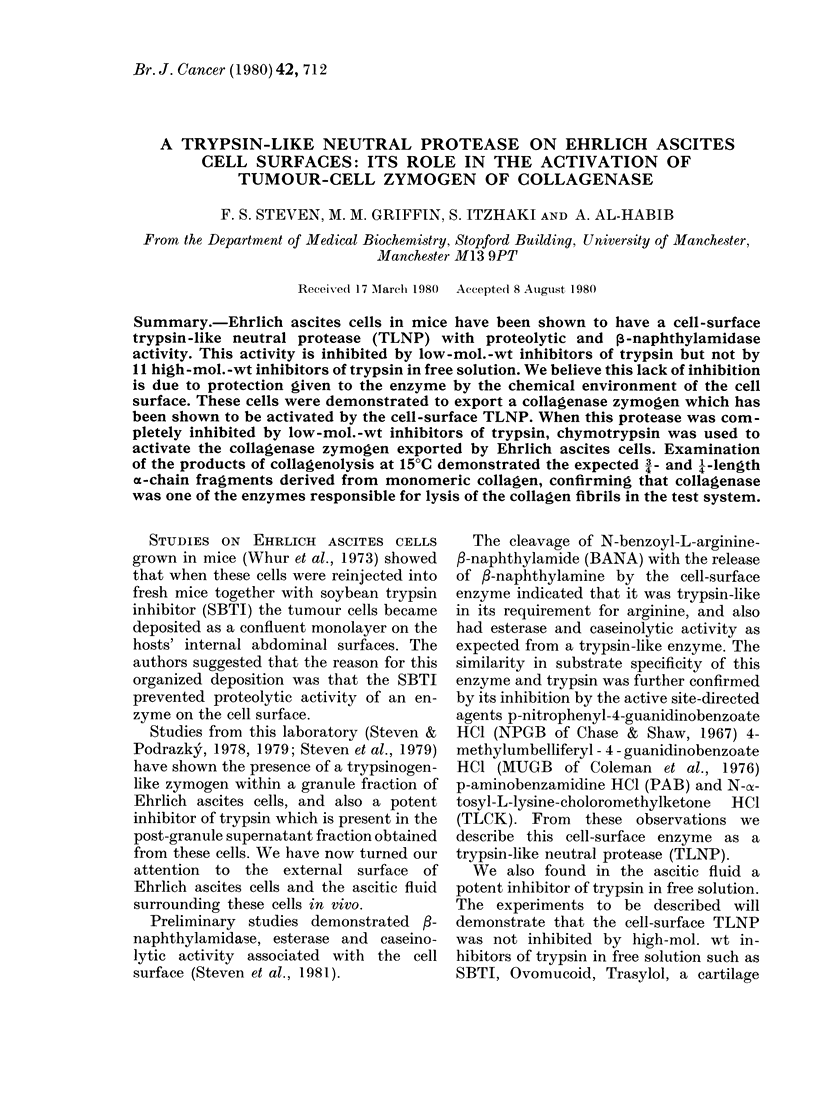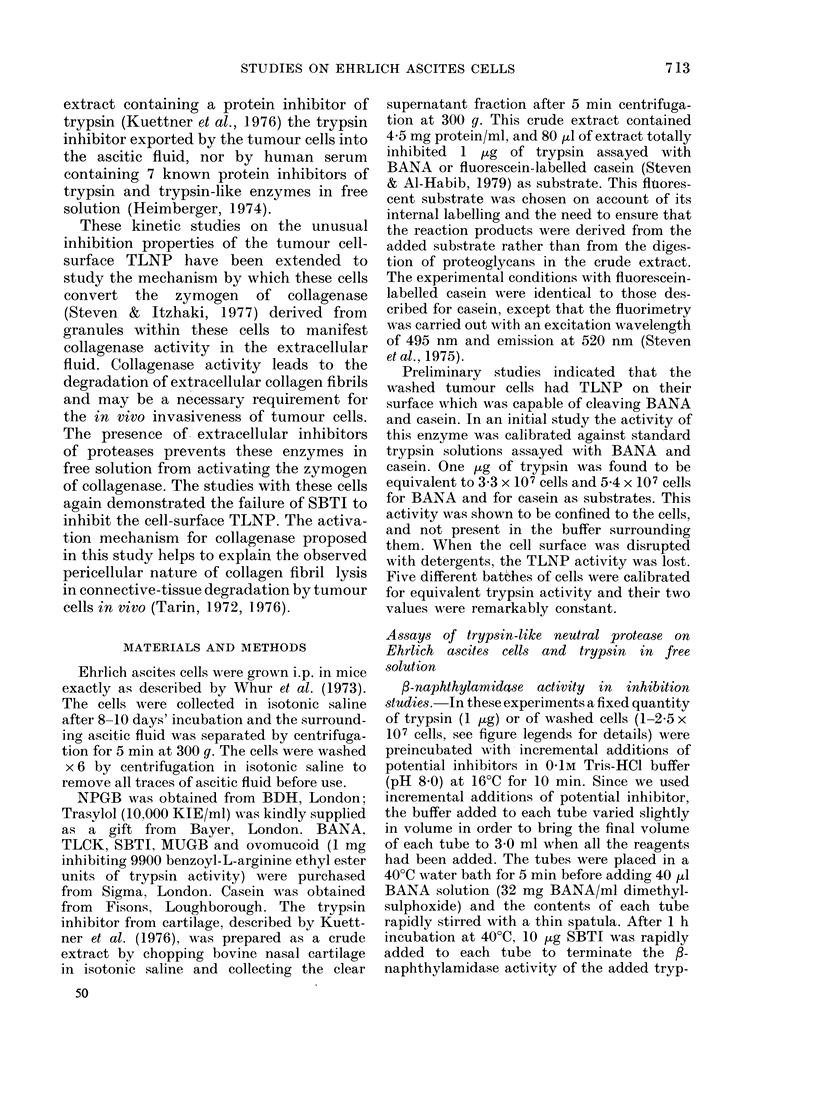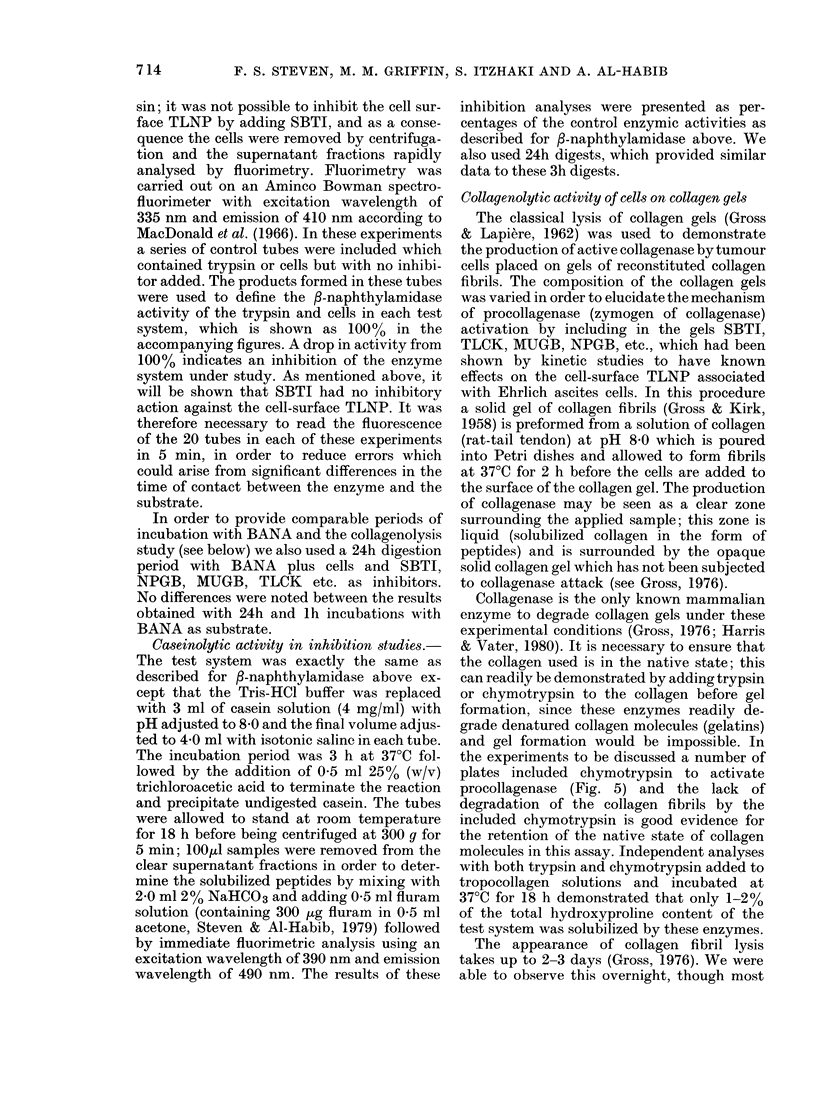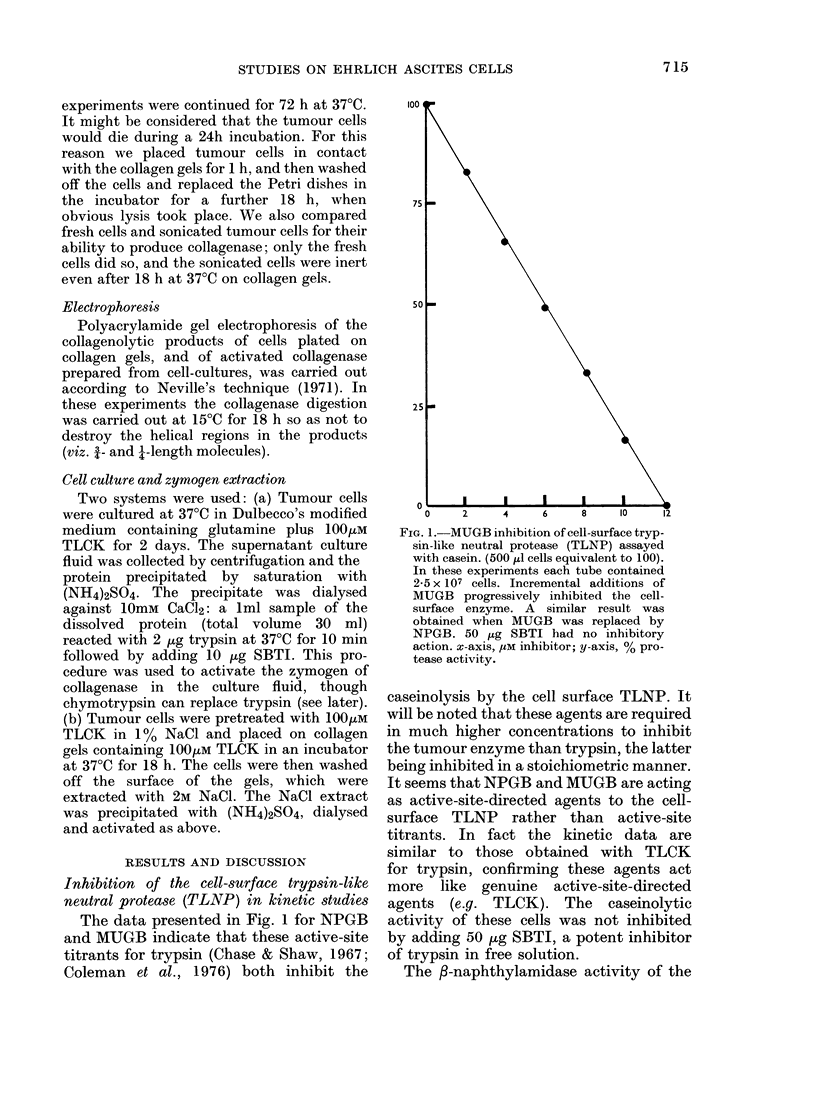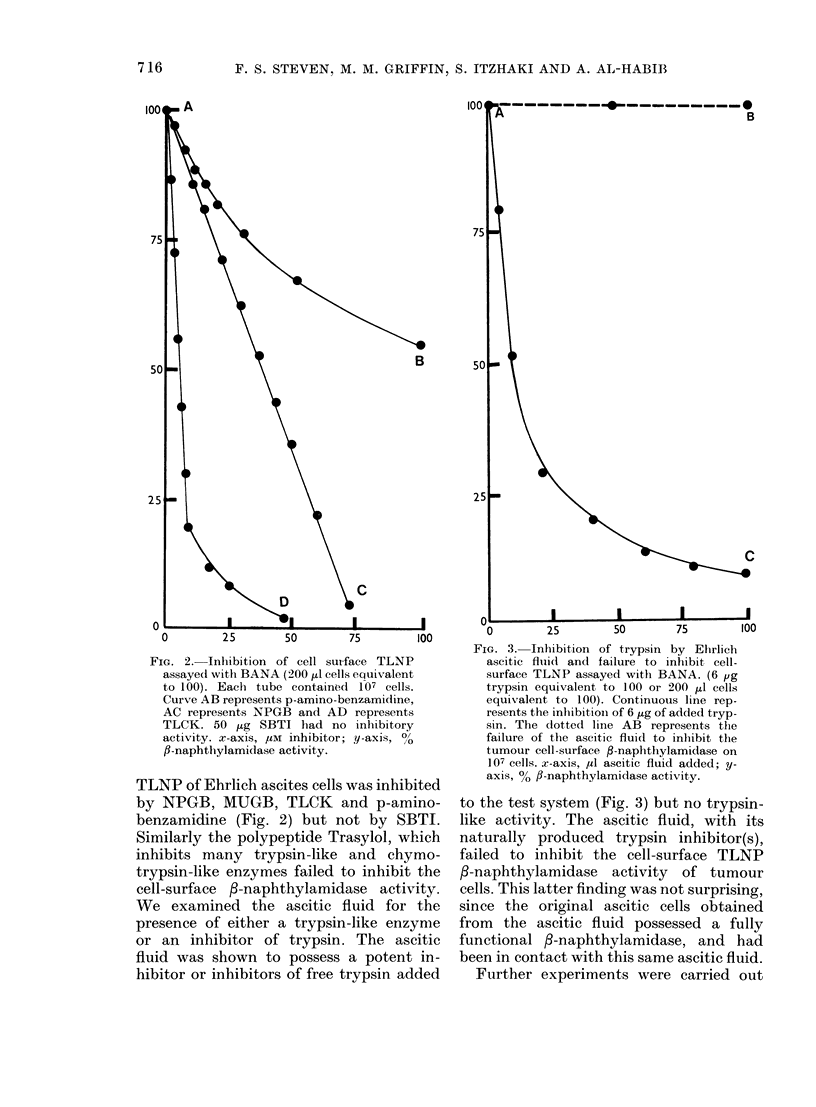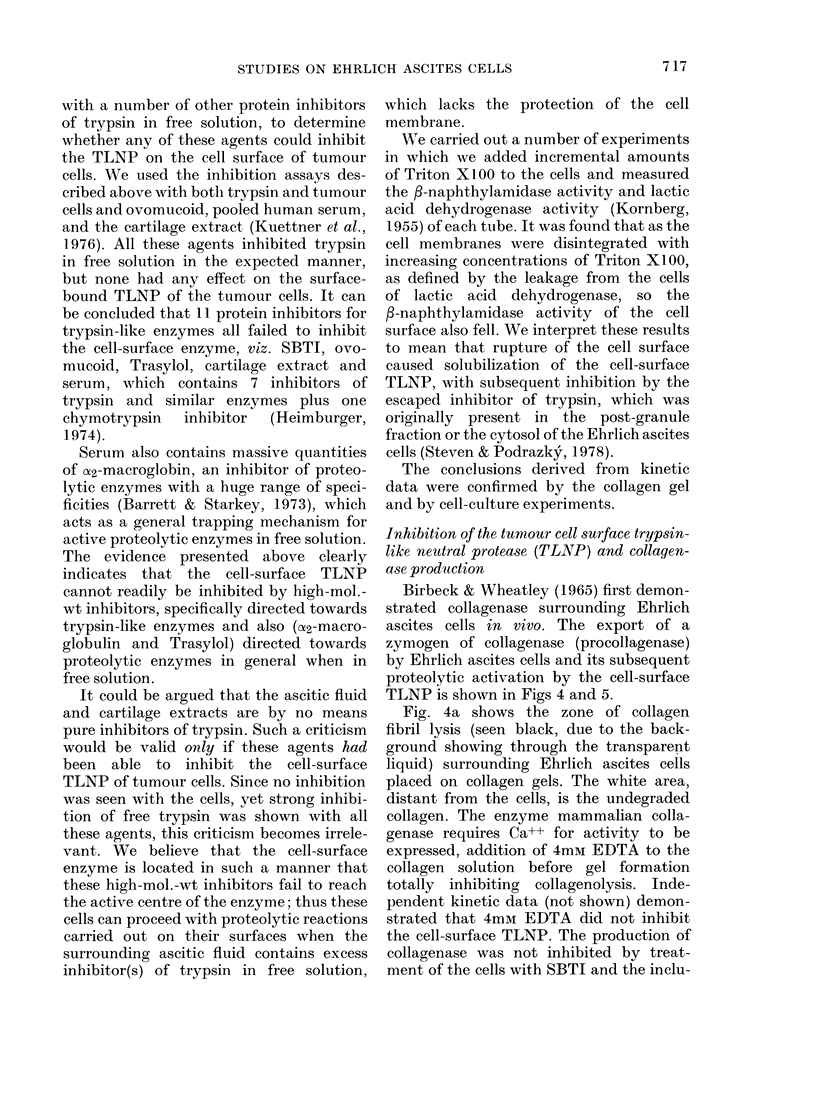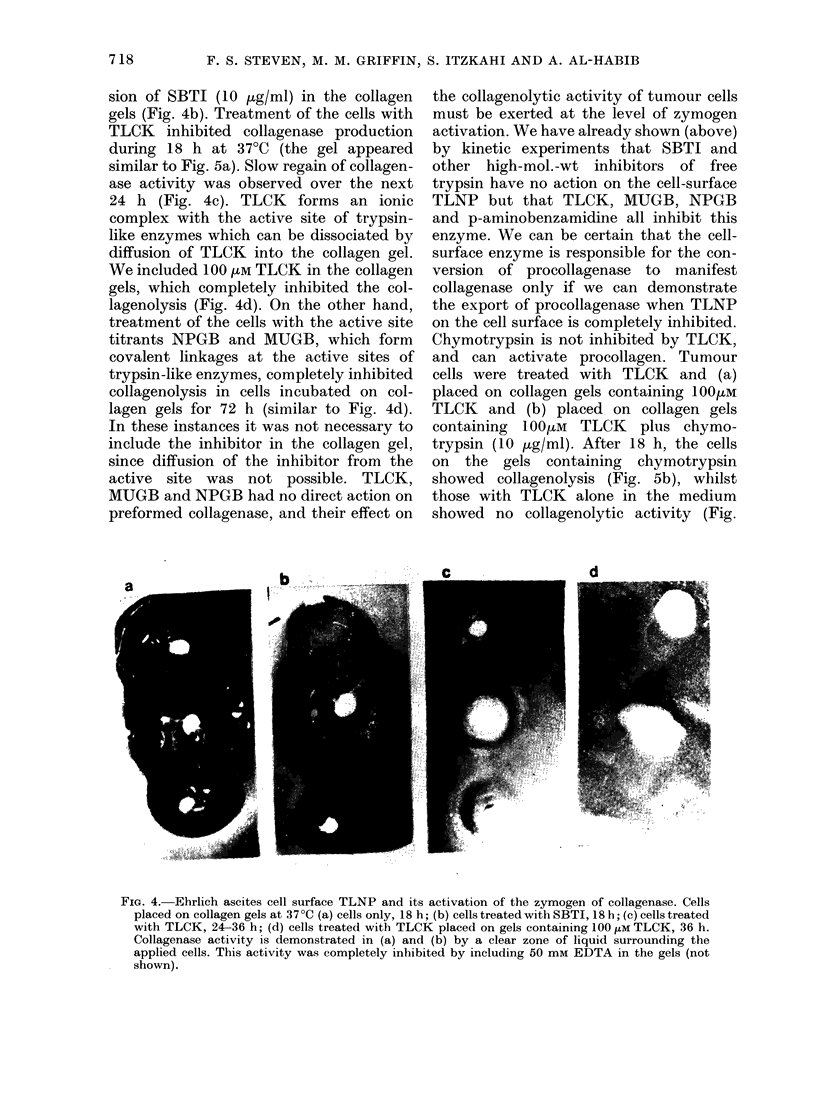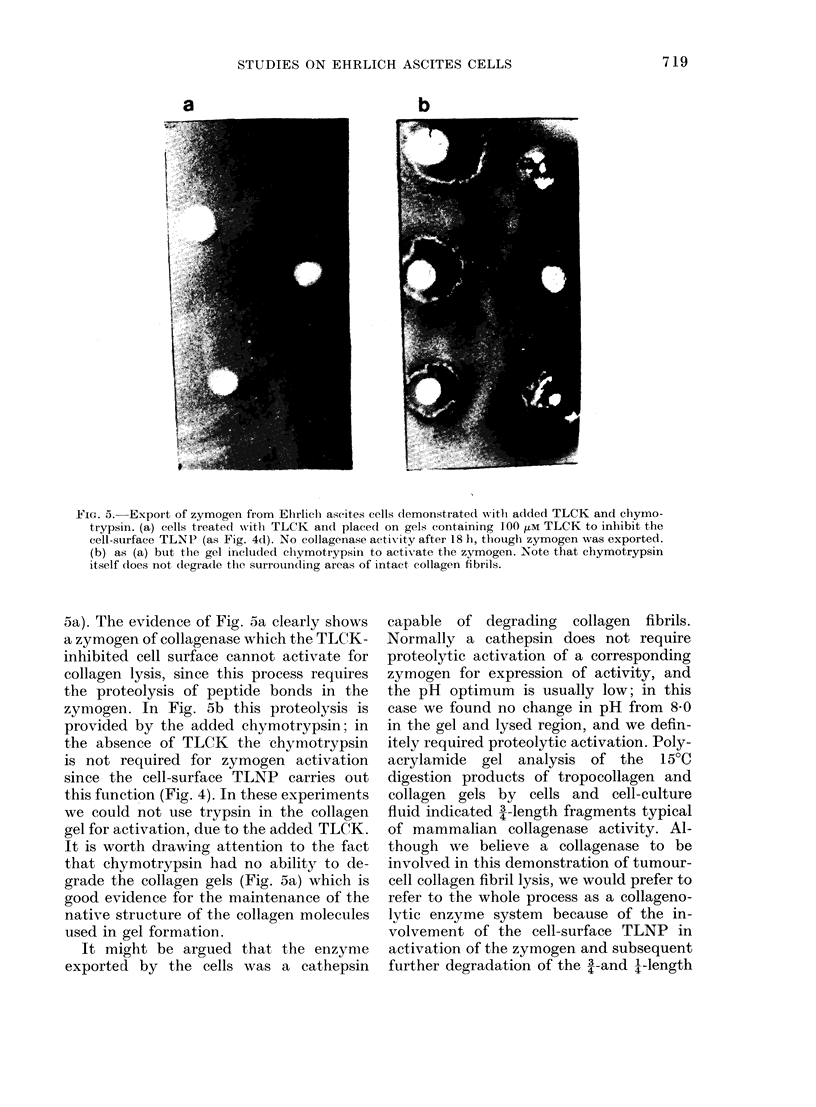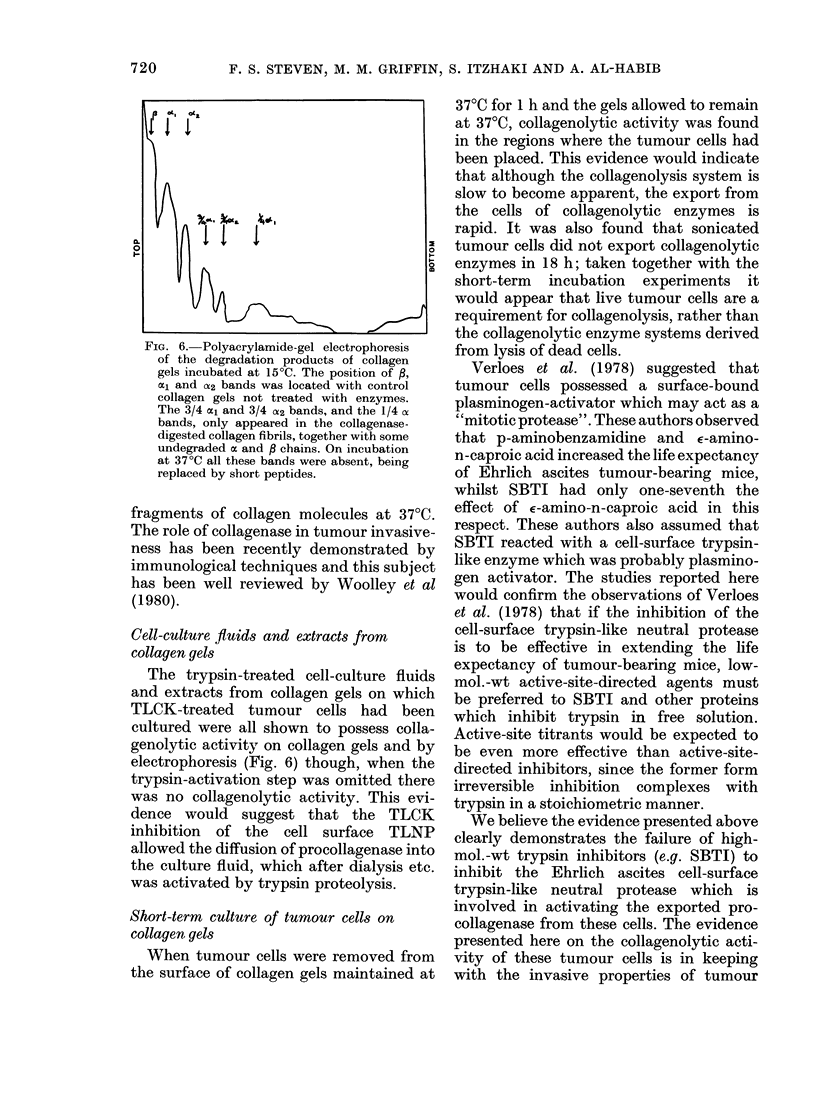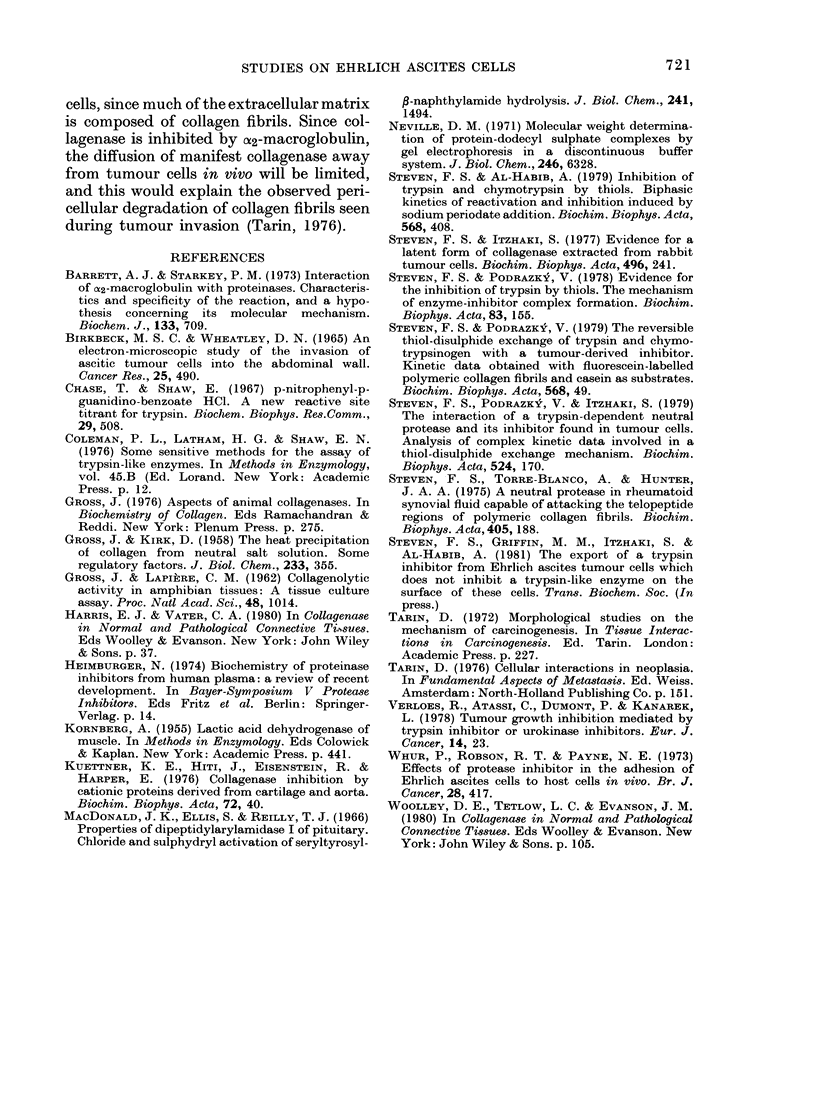# A trypsin-like neutral protease on Ehrlich ascites cell surfaces: its role in the activation of tumour-cell zymogen of collagenase.

**DOI:** 10.1038/bjc.1980.306

**Published:** 1980-11

**Authors:** F. S. Steven, M. M. Griffin, S. Itzhaki, A. Al-Habib

## Abstract

**Images:**


*Br. J. Cancer* (1980) **42**, 712

### A TRYPSIN-LIKE NEUTRAL PROTEASE ON EHRLICH ASCITES CELL SURFACES: ITS ROLE IN THE ACTIVATION OF TUMOUR-CELL ZYMOGEN OF COLLAGENASE

F. S. STEVEN, M. M. GRIFFIN, S. ITZHAKI AND A. AL-HABIB

*From the Department of Medical Biochemistry, Stopford Building, University of Manchester, Manchester M13 9PT*

Received 17 March 1980 Accepted 8 August 1980

**Summary.—Ehrlich ascites cells in mice have been shown to have a cell-surface trypsin-like neutral protease (TLNP) with proteolytic and β-naphthylamidase activity. This activity is inhibited by low-mol.-wt inhibitors of trypsin but not by 11 high-mol.-wt inhibitors of trypsin in free solution. We believe this lack of inhibition is due to protection given to the enzyme by the chemical environment of the cell surface. These cells were demonstrated to export a collagenase zymogen which has been shown to be activated by the cell-surface TLNP. When this protease was completely inhibited by low-mol.-wt inhibitors of trypsin, chymotrypsin was used to activate the collagenase zymogen exported by Ehrlich ascites cells. Examination of the products of collagenolysis at 15°C demonstrated the expected $\frac{3}{4}$- and $\frac{1}{4}$-length α-chain fragments derived from monomeric collagen, confirming that collagenase was one of the enzymes responsible for lysis of the collagen fibrils in the test system.**

STUDIES ON EHRLICH ASCITES CELLS grown in mice (Whur *et al.*, 1973) showed that when these cells were reinjected into fresh mice together with soybean trypsin inhibitor (SBTI) the tumour cells became deposited as a confluent monolayer on the hosts' internal abdominal surfaces. The authors suggested that the reason for this organized deposition was that the SBTI prevented proteolytic activity of an enzyme on the cell surface.

Studies from this laboratory (Steven & Podrazký, 1978, 1979; Steven *et al.*, 1979) have shown the presence of a trypsinogen-like zymogen within a granule fraction of Ehrlich ascites cells, and also a potent inhibitor of trypsin which is present in the post-granule supernatant fraction obtained from these cells. We have now turned our attention to the external surface of Ehrlich ascites cells and the ascitic fluid surrounding these cells *in vivo*.

Preliminary studies demonstrated β-naphthylamidase, esterase and caseinolytic activity associated with the cell surface (Steven *et al.*, 1981).

The cleavage of N-benzoyl-L-arginine-β-naphthylamide (BANA) with the release of β-naphthylamine by the cell-surface enzyme indicated that it was trypsin-like in its requirement for arginine, and also had esterase and caseinolytic activity as expected from a trypsin-like enzyme. The similarity in substrate specificity of this enzyme and trypsin was further confirmed by its inhibition by the active site-directed agents p-nitrophenyl-4-guanidinobenzoate HCl (NPGB of Chase & Shaw, 1967) 4-methylumbelliferyl-4-guanidinobenzoate HCl (MUGB of Coleman *et al.*, 1976) p-aminobenzamidine HCl (PAB) and N-α-tosyl-L-lysine-choloromethylketone HCl (TLCK). From these observations we describe this cell-surface enzyme as a trypsin-like neutral protease (TLNP).

We also found in the ascitic fluid a potent inhibitor of trypsin in free solution. The experiments to be described will demonstrate that the cell-surface TLNP was not inhibited by high-mol. wt inhibitors of trypsin in free solution such as SBTI, Ovomucoid, Trasylol, a cartilage extract containing a protein inhibitor of trypsin (Kuettner *et al.*, 1976) the trypsin inhibitor exported by the tumour cells into the ascitic fluid, nor by human serum containing 7 known protein inhibitors of trypsin and trypsin-like enzymes in free solution (Heimberger, 1974).

These kinetic studies on the unusual inhibition properties of the tumour cell-surface TLNP have been extended to study the mechanism by which these cells convert the zymogen of collagenase (Steven & Itzhaki, 1977) derived from granules within these cells to manifest collagenase activity in the extracellular fluid. Collagenase activity leads to the degradation of extracellular collagen fibrils and may be a necessary requirement for the *in vivo* invasiveness of tumour cells. The presence of extracellular inhibitors of proteases prevents these enzymes in free solution from activating the zymogen of collagenase. The studies with these cells again demonstrated the failure of SBTI to inhibit the cell-surface TLNP. The activation mechanism for collagenase proposed in this study helps to explain the observed pericellular nature of collagen fibril lysis in connective-tissue degradation by tumour cells *in vivo* (Tarin, 1972, 1976).

### MATERIALS AND METHODS

Ehrlich ascites cells were grown i.p. in mice exactly as described by Whur *et al.* (1973). The cells were collected in isotonic saline after 8–10 days' incubation and the surrounding ascitic fluid was separated by centrifugation for 5 min at 300 *g*. The cells were washed ×6 by centrifugation in isotonic saline to remove all traces of ascitic fluid before use.

NPGB was obtained from BDH, London; Trasylol (10,000 KIE/ml) was kindly supplied as a gift from Bayer, London. BANA, TLCK, SBTI, MUGB and ovomucoid (1 mg inhibiting 9900 benzoyl-L-arginine ethyl ester units of trypsin activity) were purchased from Sigma, London. Casein was obtained from Fisons, Loughborough. The trypsin inhibitor from cartilage, described by Kuettner *et al.* (1976), was prepared as a crude extract by chopping bovine nasal cartilage in isotonic saline and collecting the clear supernatant fraction after 5 min centrifugation at 300 *g*. This crude extract contained 4·5 mg protein/ml, and 80 μl of extract totally inhibited 1 μg of trypsin assayed with BANA or fluorescein-labelled casein (Steven & Al-Habib, 1979) as substrate. This fluorescent substrate was chosen on account of its internal labelling and the need to ensure that the reaction products were derived from the added substrate rather than from the digestion of proteoglycans in the crude extract. The experimental conditions with fluorescein-labelled casein were identical to those described for casein, except that the fluorimetry was carried out with an excitation wavelength of 495 nm and emission at 520 nm (Steven *et al.*, 1975).

Preliminary studies indicated that the washed tumour cells had TLNP on their surface which was capable of cleaving BANA and casein. In an initial study the activity of this enzyme was calibrated against standard trypsin solutions assayed with BANA and casein. One μg of trypsin was found to be equivalent to $3{\cdot}3 \times 10^7$ cells and $5{\cdot}4 \times 10^7$ cells for BANA and for casein as substrates. This activity was shown to be confined to the cells, and not present in the buffer surrounding them. When the cell surface was disrupted with detergents, the TLNP activity was lost. Five different batches of cells were calibrated for equivalent trypsin activity and their two values were remarkably constant.

### *Assays of trypsin-like neutral protease on Ehrlich ascites cells and trypsin in free solution*

*β-naphthylamidase activity in inhibition studies.*—In these experiments a fixed quantity of trypsin (1 μg) or of washed cells ($1\text{–}2{\cdot}5 \times 10^7$ cells, see figure legends for details) were preincubated with incremental additions of potential inhibitors in 0·1M Tris-HCl buffer (pH 8·0) at 16°C for 10 min. Since we used incremental additions of potential inhibitor, the buffer added to each tube varied slightly in volume in order to bring the final volume of each tube to 3·0 ml when all the reagents had been added. The tubes were placed in a 40°C water bath for 5 min before adding 40 μl BANA solution (32 mg BANA/ml dimethylsulphoxide) and the contents of each tube rapidly stirred with a thin spatula. After 1 h incubation at 40°C, 10 μg SBTI was rapidly added to each tube to terminate the β-naphthylamidase activity of the added trypsin; it was not possible to inhibit the cell surface TLNP by adding SBTI, and as a consequence the cells were removed by centrifugation and the supernatant fractions rapidly analysed by fluorimetry. Fluorimetry was carried out on an Aminco Bowman spectrofluorimeter with excitation wavelength of 335 nm and emission of 410 nm according to MacDonald *et al.* (1966). In these experiments a series of control tubes were included which contained trypsin or cells but with no inhibitor added. The products formed in these tubes were used to define the β-naphthylamidase activity of the trypsin and cells in each test system, which is shown as 100% in the accompanying figures. A drop in activity from 100% indicates an inhibition of the enzyme system under study. As mentioned above, it will be shown that SBTI had no inhibitory action against the cell-surface TLNP. It was therefore necessary to read the fluorescence of the 20 tubes in each of these experiments in 5 min, in order to reduce errors which could arise from significant differences in the time of contact between the enzyme and the substrate.

In order to provide comparable periods of incubation with BANA and the collagenolysis study (see below) we also used a 24h digestion period with BANA plus cells and SBTI, NPGB, MUGB, TLCK etc. as inhibitors. No differences were noted between the results obtained with 24h and 1h incubations with BANA as substrate.

*Caseinolytic activity in inhibition studies.*— The test system was exactly the same as described for β-naphthylamidase above except that the Tris-HCl buffer was replaced with 3 ml of casein solution (4 mg/ml) with pH adjusted to 8·0 and the final volume adjusted to 4·0 ml with isotonic saline in each tube. The incubation period was 3 h at 37°C followed by the addition of 0·5 ml 25% (w/v) trichloroacetic acid to terminate the reaction and precipitate undigested casein. The tubes were allowed to stand at room temperature for 18 h before being centrifuged at 300 *g* for 5 min; 100μl samples were removed from the clear supernatant fractions in order to determine the solubilized peptides by mixing with 2·0 ml 2% $NaHCO_3$ and adding 0·5 ml fluram solution (containing 300 μg fluram in 0·5 ml acetone, Steven & Al-Habib, 1979) followed by immediate fluorimetric analysis using an excitation wavelength of 390 nm and emission wavelength of 490 nm. The results of these inhibition analyses were presented as percentages of the control enzymic activities as described for β-naphthylamidase above. We also used 24h digests, which provided similar data to these 3h digests.

*Collagenolytic activity of cells on collagen gels*

The classical lysis of collagen gels (Gross & Lapière, 1962) was used to demonstrate the production of active collagenase by tumour cells placed on gels of reconstituted collagen fibrils. The composition of the collagen gels was varied in order to elucidate the mechanism of procollagenase (zymogen of collagenase) activation by including in the gels SBTI, TLCK, MUGB, NPGB, etc., which had been shown by kinetic studies to have known effects on the cell-surface TLNP associated with Ehrlich ascites cells. In this procedure a solid gel of collagen fibrils (Gross & Kirk, 1958) is preformed from a solution of collagen (rat-tail tendon) at pH 8·0 which is poured into Petri dishes and allowed to form fibrils at 37°C for 2 h before the cells are added to the surface of the collagen gel. The production of collagenase may be seen as a clear zone surrounding the applied sample; this zone is liquid (solubilized collagen in the form of peptides) and is surrounded by the opaque solid collagen gel which has not been subjected to collagenase attack (see Gross, 1976).

Collagenase is the only known mammalian enzyme to degrade collagen gels under these experimental conditions (Gross, 1976; Harris & Vater, 1980). It is necessary to ensure that the collagen used is in the native state; this can readily be demonstrated by adding trypsin or chymotrypsin to the collagen before gel formation, since these enzymes readily degrade denatured collagen molecules (gelatins) and gel formation would be impossible. In the experiments to be discussed a number of plates included chymotrypsin to activate procollagenase (Fig. 5) and the lack of degradation of the collagen fibrils by the included chymotrypsin is good evidence for the retention of the native state of collagen molecules in this assay. Independent analyses with both trypsin and chymotrypsin added to tropocollagen solutions and incubated at 37°C for 18 h demonstrated that only 1–2% of the total hydroxyproline content of the test system was solubilized by these enzymes.

The appearance of collagen fibril lysis takes up to 2–3 days (Gross, 1976). We were able to observe this overnight, though most experiments were continued for 72 h at 37°C. It might be considered that the tumour cells would die during a 24h incubation. For this reason we placed tumour cells in contact with the collagen gels for 1 h, and then washed off the cells and replaced the Petri dishes in the incubator for a further 18 h, when obvious lysis took place. We also compared fresh cells and sonicated tumour cells for their ability to produce collagenase; only the fresh cells did so, and the sonicated cells were inert even after 18 h at 37°C on collagen gels.

*Electrophoresis*

Polyacrylamide gel electrophoresis of the collagenolytic products of cells plated on collagen gels, and of activated collagenase prepared from cell-cultures, was carried out according to Neville's technique (1971). In these experiments the collagenase digestion was carried out at 15°C for 18 h so as not to destroy the helical regions in the products (*viz.* $\frac{3}{4}$- and $\frac{1}{4}$-length molecules).

*Cell culture and zymogen extraction*

Two systems were used: (a) Tumour cells were cultured at 37°C in Dulbecco's modified medium containing glutamine plus 100$\mu$M TLCK for 2 days. The supernatant culture fluid was collected by centrifugation and the protein precipitated by saturation with $(NH_4)_2SO_4$. The precipitate was dialysed against 10mM $CaCl_2$: a 1ml sample of the dissolved protein (total volume 30 ml) reacted with 2 $\mu$g trypsin at 37°C for 10 min followed by adding 10 $\mu$g SBTI. This procedure was used to activate the zymogen of collagenase in the culture fluid, though chymotrypsin can replace trypsin (see later). (b) Tumour cells were pretreated with 100$\mu$M TLCK in 1% NaCl and placed on collagen gels containing 100$\mu$M TLCK in an incubator at 37°C for 18 h. The cells were then washed off the surface of the gels, which were extracted with 2M NaCl. The NaCl extract was precipitated with $(NH_4)_2SO_4$, dialysed and activated as above.

### RESULTS AND DISCUSSION

*Inhibition of the cell-surface trypsin-like neutral protease (TLNP) in kinetic studies*

The data presented in Fig. 1 for NPGB and MUGB indicate that these active-site titrants for trypsin (Chase & Shaw, 1967; Coleman *et al.*, 1976) both inhibit the caseinolysis by the cell surface TLNP. It will be noted that these agents are required in much higher concentrations to inhibit the tumour enzyme than trypsin, the latter being inhibited in a stoichiometric manner. It seems that NPGB and MUGB are acting as active-site-directed agents to the cell-surface TLNP rather than active-site titrants. In fact the kinetic data are similar to those obtained with TLCK for trypsin, confirming these agents act more like genuine active-site-directed agents (*e.g.* TLCK). The caseinolytic activity of these cells was not inhibited by adding 50 $\mu$g SBTI, a potent inhibitor of trypsin in free solution.

FIG. 1.—MUGB inhibition of cell-surface trypsin-like neutral protease (TLNP) assayed with casein. (500 $\mu$l cells equivalent to 100). In these experiments each tube contained $2{\cdot}5 \times 10^7$ cells. Incremental additions of MUGB progressively inhibited the cell-surface enzyme. A similar result was obtained when MUGB was replaced by NPGB. 50 $\mu$g SBTI had no inhibitory action. $x$-axis, $\mu$M inhibitor; $y$-axis, % protease activity.

The $\beta$-naphthylamidase activity of the

FIG. 2.—Inhibition of cell surface TLNP assayed with BANA (200 μl cells equivalent to 100). Each tube contained $10^7$ cells. Curve AB represents p-amino-benzamidine, AC represents NPGB and AD represents TLCK. 50 μg SBTI had no inhibitory activity. $x$-axis, μM inhibitor; $y$-axis, % β-naphthylamidase activity.

FIG. 3.—Inhibition of trypsin by Ehrlich ascitic fluid and failure to inhibit cell-surface TLNP assayed with BANA. (6 μg trypsin equivalent to 100 or 200 μl cells equivalent to 100). Continuous line represents the inhibition of 6 μg of added trypsin. The dotted line AB represents the failure of the ascitic fluid to inhibit the tumour cell-surface β-naphthylamidase on $10^7$ cells. $x$-axis, μl ascitic fluid added; $y$-axis, % β-naphthylamidase activity.

TLNP of Ehrlich ascites cells was inhibited by NPGB, MUGB, TLCK and p-amino-benzamidine (Fig. 2) but not by SBTI. Similarly the polypeptide Trasylol, which inhibits many trypsin-like and chymo-trypsin-like enzymes failed to inhibit the cell-surface β-naphthylamidase activity. We examined the ascitic fluid for the presence of either a trypsin-like enzyme or an inhibitor of trypsin. The ascitic fluid was shown to possess a potent inhibitor or inhibitors of free trypsin added to the test system (Fig. 3) but no trypsin-like activity. The ascitic fluid, with its naturally produced trypsin inhibitor(s), failed to inhibit the cell-surface TLNP β-naphthylamidase activity of tumour cells. This latter finding was not surprising, since the original ascitic cells obtained from the ascitic fluid possessed a fully functional β-naphthylamidase, and had been in contact with this same ascitic fluid.

Further experiments were carried out with a number of other protein inhibitors of trypsin in free solution, to determine whether any of these agents could inhibit the TLNP on the cell surface of tumour cells. We used the inhibition assays described above with both trypsin and tumour cells and ovomucoid, pooled human serum, and the cartilage extract (Kuettner *et al.*, 1976). All these agents inhibited trypsin in free solution in the expected manner, but none had any effect on the surface-bound TLNP of the tumour cells. It can be concluded that 11 protein inhibitors for trypsin-like enzymes all failed to inhibit the cell-surface enzyme, *viz.* SBTI, ovomucoid, Trasylol, cartilage extract and serum, which contains 7 inhibitors of trypsin and similar enzymes plus one chymotrypsin inhibitor (Heimburger, 1974).

Serum also contains massive quantities of $\alpha_2$-macroglobin, an inhibitor of proteolytic enzymes with a huge range of specificities (Barrett & Starkey, 1973), which acts as a general trapping mechanism for active proteolytic enzymes in free solution. The evidence presented above clearly indicates that the cell-surface TLNP cannot readily be inhibited by high-mol.-wt inhibitors, specifically directed towards trypsin-like enzymes and also ($\alpha_2$-macroglobulin and Trasylol) directed towards proteolytic enzymes in general when in free solution.

It could be argued that the ascitic fluid and cartilage extracts are by no means pure inhibitors of trypsin. Such a criticism would be valid *only* if these agents *had* been able to inhibit the cell-surface TLNP of tumour cells. Since no inhibition was seen with the cells, yet strong inhibition of free trypsin was shown with all these agents, this criticism becomes irrelevant. We believe that the cell-surface enzyme is located in such a manner that these high-mol.-wt inhibitors fail to reach the active centre of the enzyme; thus these cells can proceed with proteolytic reactions carried out on their surfaces when the surrounding ascitic fluid contains excess inhibitor(s) of trypsin in free solution, which lacks the protection of the cell membrane.

We carried out a number of experiments in which we added incremental amounts of Triton X100 to the cells and measured the $\beta$-naphthylamidase activity and lactic acid dehydrogenase activity (Kornberg, 1955) of each tube. It was found that as the cell membranes were disintegrated with increasing concentrations of Triton X100, as defined by the leakage from the cells of lactic acid dehydrogenase, so the $\beta$-naphthylamidase activity of the cell surface also fell. We interpret these results to mean that rupture of the cell surface caused solubilization of the cell-surface TLNP, with subsequent inhibition by the escaped inhibitor of trypsin, which was originally present in the post-granule fraction or the cytosol of the Ehrlich ascites cells (Steven & Podrazký, 1978).

The conclusions derived from kinetic data were confirmed by the collagen gel and by cell-culture experiments.

*Inhibition of the tumour cell surface trypsin-like neutral protease (TLNP) and collagenase production*

Birbeck & Wheatley (1965) first demonstrated collagenase surrounding Ehrlich ascites cells *in vivo*. The export of a zymogen of collagenase (procollagenase) by Ehrlich ascites cells and its subsequent proteolytic activation by the cell-surface TLNP is shown in Figs 4 and 5.

Fig. 4a shows the zone of collagen fibril lysis (seen black, due to the background showing through the transparent liquid) surrounding Ehrlich ascites cells placed on collagen gels. The white area, distant from the cells, is the undegraded collagen. The enzyme mammalian collagenase requires $Ca^{++}$ for activity to be expressed, addition of 4mM EDTA to the collagen solution before gel formation totally inhibiting collagenolysis. Independent kinetic data (not shown) demonstrated that 4mM EDTA did not inhibit the cell-surface TLNP. The production of collagenase was not inhibited by treatment of the cells with SBTI and the inclusion of SBTI (10 μg/ml) in the collagen gels (Fig. 4b). Treatment of the cells with TLCK inhibited collagenase production during 18 h at 37°C (the gel appeared similar to Fig. 5a). Slow regain of collagenase activity was observed over the next 24 h (Fig. 4c). TLCK forms an ionic complex with the active site of trypsin-like enzymes which can be dissociated by diffusion of TLCK into the collagen gel. We included 100 μM TLCK in the collagen gels, which completely inhibited the collagenolysis (Fig. 4d). On the other hand, treatment of the cells with the active site titrants NPGB and MUGB, which form covalent linkages at the active sites of trypsin-like enzymes, completely inhibited collagenolysis in cells incubated on collagen gels for 72 h (similar to Fig. 4d). In these instances it was not necessary to include the inhibitor in the collagen gel, since diffusion of the inhibitor from the active site was not possible. TLCK, MUGB and NPGB had no direct action on preformed collagenase, and their effect on the collagenolytic activity of tumour cells must be exerted at the level of zymogen activation. We have already shown (above) by kinetic experiments that SBTI and other high-mol.-wt inhibitors of free trypsin have no action on the cell-surface TLNP but that TLCK, MUGB, NPGB and p-aminobenzamidine all inhibit this enzyme. We can be certain that the cell-surface enzyme is responsible for the conversion of procollagenase to manifest collagenase only if we can demonstrate the export of procollagenase when TLNP on the cell surface is completely inhibited. Chymotrypsin is not inhibited by TLCK, and can activate procollagen. Tumour cells were treated with TLCK and (a) placed on collagen gels containing 100μM TLCK and (b) placed on collagen gels containing 100μM TLCK plus chymotrypsin (10 μg/ml). After 18 h, the cells on the gels containing chymotrypsin showed collagenolysis (Fig. 5b), whilst those with TLCK alone in the medium showed no collagenolytic activity (Fig.

FIG. 4.—Ehrlich ascites cell surface TLNP and its activation of the zymogen of collagenase. Cells placed on collagen gels at 37°C (a) cells only, 18 h; (b) cells treated with SBTI, 18 h; (c) cells treated with TLCK, 24–36 h; (d) cells treated with TLCK placed on gels containing 100 μM TLCK, 36 h. Collagenase activity is demonstrated in (a) and (b) by a clear zone of liquid surrounding the applied cells. This activity was completely inhibited by including 50 mM EDTA in the gels (not shown).

Fig. 5.—Export of zymogen from Ehrlich ascites cells demonstrated with added TLCK and chymotrypsin. (a) cells treated with TLCK and placed on gels containing 100 μM TLCK to inhibit the cell-surface TLNP (as Fig. 4d). No collagenase activity after 18 h, though zymogen was exported. (b) as (a) but the gel included chymotrypsin to activate the zymogen. Note that chymotrypsin itself does not degrade the surrounding areas of intact collagen fibrils.

5a). The evidence of Fig. 5a clearly shows a zymogen of collagenase which the TLCK-inhibited cell surface cannot activate for collagen lysis, since this process requires the proteolysis of peptide bonds in the zymogen. In Fig. 5b this proteolysis is provided by the added chymotrypsin; in the absence of TLCK the chymotrypsin is not required for zymogen activation since the cell-surface TLNP carries out this function (Fig. 4). In these experiments we could not use trypsin in the collagen gel for activation, due to the added TLCK. It is worth drawing attention to the fact that chymotrypsin had no ability to degrade the collagen gels (Fig. 5a) which is good evidence for the maintenance of the native structure of the collagen molecules used in gel formation.

It might be argued that the enzyme exported by the cells was a cathepsin capable of degrading collagen fibrils. Normally a cathepsin does not require proteolytic activation of a corresponding zymogen for expression of activity, and the pH optimum is usually low; in this case we found no change in pH from 8·0 in the gel and lysed region, and we definitely required proteolytic activation. Polyacrylamide gel analysis of the 15°C digestion products of tropocollagen and collagen gels by cells and cell-culture fluid indicated $\frac{3}{4}$-length fragments typical of mammalian collagenase activity. Although we believe a collagenase to be involved in this demonstration of tumour-cell collagen fibril lysis, we would prefer to refer to the whole process as a collagenolytic enzyme system because of the involvement of the cell-surface TLNP in activation of the zymogen and subsequent further degradation of the $\frac{3}{4}$-and $\frac{1}{4}$-length

FIG. 6.—Polyacrylamide-gel electrophoresis of the degradation products of collagen gels incubated at 15°C. The position of $\beta$, $\alpha_1$ and $\alpha_2$ bands was located with control collagen gels not treated with enzymes. The 3/4 $\alpha_1$ and 3/4 $\alpha_2$ bands, and the 1/4 $\alpha$ bands, only appeared in the collagenase-digested collagen fibrils, together with some undegraded $\alpha$ and $\beta$ chains. On incubation at 37°C all these bands were absent, being replaced by short peptides.

fragments of collagen molecules at 37°C. The role of collagenase in tumour invasiveness has been recently demonstrated by immunological techniques and this subject has been well reviewed by Woolley *et al* (1980).

*Cell-culture fluids and extracts from collagen gels*

The trypsin-treated cell-culture fluids and extracts from collagen gels on which TLCK-treated tumour cells had been cultured were all shown to possess collagenolytic activity on collagen gels and by electrophoresis (Fig. 6) though, when the trypsin-activation step was omitted there was no collagenolytic activity. This evidence would suggest that the TLCK inhibition of the cell surface TLNP allowed the diffusion of procollagenase into the culture fluid, which after dialysis etc. was activated by trypsin proteolysis.

*Short-term culture of tumour cells on collagen gels*

When tumour cells were removed from the surface of collagen gels maintained at 37°C for 1 h and the gels allowed to remain at 37°C, collagenolytic activity was found in the regions where the tumour cells had been placed. This evidence would indicate that although the collagenolysis system is slow to become apparent, the export from the cells of collagenolytic enzymes is rapid. It was also found that sonicated tumour cells did not export collagenolytic enzymes in 18 h; taken together with the short-term incubation experiments it would appear that live tumour cells are a requirement for collagenolysis, rather than the collagenolytic enzyme systems derived from lysis of dead cells.

Verloes *et al.* (1978) suggested that tumour cells possessed a surface-bound plasminogen-activator which may act as a "mitotic protease". These authors observed that p-aminobenzamidine and $\epsilon$-amino-n-caproic acid increased the life expectancy of Ehrlich ascites tumour-bearing mice, whilst SBTI had only one-seventh the effect of $\epsilon$-amino-n-caproic acid in this respect. These authors also assumed that SBTI reacted with a cell-surface trypsin-like enzyme which was probably plasminogen activator. The studies reported here would confirm the observations of Verloes *et al.* (1978) that if the inhibition of the cell-surface trypsin-like neutral protease is to be effective in extending the life expectancy of tumour-bearing mice, low-mol.-wt active-site-directed agents must be preferred to SBTI and other proteins which inhibit trypsin in free solution. Active-site titrants would be expected to be even more effective than active-site-directed inhibitors, since the former form irreversible inhibition complexes with trypsin in a stoichiometric manner.

We believe the evidence presented above clearly demonstrates the failure of high-mol.-wt trypsin inhibitors (*e.g.* SBTI) to inhibit the Ehrlich ascites cell-surface trypsin-like neutral protease which is involved in activating the exported procollagenase from these cells. The evidence presented here on the collagenolytic activity of these tumour cells is in keeping with the invasive properties of tumour cells, since much of the extracellular matrix is composed of collagen fibrils. Since collagenase is inhibited by $\alpha_2$-macroglobulin, the diffusion of manifest collagenase away from tumour cells *in vivo* will be limited, and this would explain the observed pericellular degradation of collagen fibrils seen during tumour invasion (Tarin, 1976).

### REFERENCES

BARRETT, A. J. & STARKEY, P. M. (1973) Interaction of $\alpha_2$-macroglobulin with proteinases. Characteristics and specificity of the reaction, and a hypothesis concerning its molecular mechanism. *Biochem. J.*, **133**, 709.

BIRKBECK, M. S. C. & WHEATLEY, D. N. (1965) An electron-microscopic study of the invasion of ascitic tumour cells into the abdominal wall. *Cancer Res.*, **25**, 490.

CHASE, T. & SHAW, E. (1967) p-nitrophenyl-p-guanidino-benzoate HCl. A new reactive site titrant for trypsin. *Biochem. Biophys. Res.Comm.*, **29**, 508.

COLEMAN, P. L., LATHAM, H. G. & SHAW, E. N. (1976) Some sensitive methods for the assay of trypsin-like enzymes. In *Methods in Enzymology*, vol. 45.B (Ed. Lorand. New York: Academic Press. p. 12.

GROSS, J. (1976) Aspects of animal collagenases. In *Biochemistry of Collagen*. Eds Ramachandran & Reddi. New York: Plenum Press. p. 275.

GROSS, J. & KIRK, D. (1958) The heat precipitation of collagen from neutral salt solution. Some regulatory factors. *J. Biol. Chem.*, **233**, 355.

GROSS, J. & LAPIÈRE, C. M. (1962) Collagenolytic activity in amphibian tissues: A tissue culture assay. *Proc. Natl Acad. Sci.*, **48**, 1014.

HARRIS, E. J. & VATER, C. A. (1980) In *Collagenase in Normal and Pathological Connective Tissues*. Eds Woolley & Evanson. New York: John Wiley & Sons. p. 37.

HEIMBURGER, N. (1974) Biochemistry of proteinase inhibitors from human plasma: a review of recent development. In *Bayer-Symposium V Protease Inhibitors*. Eds Fritz *et al.* Berlin: Springer-Verlag. p. 14.

KORNBERG, A. (1955) Lactic acid dehydrogenase of muscle. In *Methods in Enzymology*. Eds Colowick & Kaplan. New York: Academic Press. p. 441.

KUETTNER, K. E., HITI, J., EISENSTEIN, R. & HARPER, E. (1976) Collagenase inhibition by cationic proteins derived from cartilage and aorta. *Biochim. Biophys. Acta*, **72**, 40.

MACDONALD, J. K., ELLIS, S. & REILLY, T. J. (1966) Properties of dipeptidylarylamidase I of pituitary. Chloride and sulphydryl activation of seryltyrosyl-$\beta$-naphthylamide hydrolysis. *J. Biol. Chem.*, **241**, 1494.

NEVILLE, D. M. (1971) Molecular weight determination of protein-dodecyl sulphate complexes by gel electrophoresis in a discontinuous buffer system. *J. Biol. Chem.*, **246**, 6328.

STEVEN, F. S. & AL-HABIB, A. (1979) Inhibition of trypsin and chymotrypsin by thiols. Biphasic kinetics of reactivation and inhibition induced by sodium periodate addition. *Biochim. Biophys. Acta*, **568**, 408.

STEVEN, F. S. & ITZHAKI, S. (1977) Evidence for a latent form of collagenase extracted from rabbit tumour cells. *Biochim. Biophys. Acta*, **496**, 241.

STEVEN, F. S. & PODRAZKÝ, V. (1978) Evidence for the inhibition of trypsin by thiols. The mechanism of enzyme-inhibitor complex formation. *Biochim. Biophys. Acta*, **83**, 155.

STEVEN, F. S. & PODRAZKÝ, V. (1979) The reversible thiol-disulphide exchange of trypsin and chymotrypsinogen with a tumour-derived inhibitor. Kinetic data obtained with fluorescein-labelled polymeric collagen fibrils and casein as substrates. *Biochim. Biophys. Acta*, **568**, 49.

STEVEN, F. S., PODRAZKÝ, V. & ITZHAKI, S. (1979) The interaction of a trypsin-dependent neutral protease and its inhibitor found in tumour cells. Analysis of complex kinetic data involved in a thiol-disulphide exchange mechanism. *Biochim. Biophys. Acta*, **524**, 170.

STEVEN, F. S., TORRE-BLANCO, A. & HUNTER, J. A. A. (1975) A neutral protease in rheumatoid synovial fluid capable of attacking the telopeptide regions of polymeric collagen fibrils. *Biochim. Biophys. Acta*, **405**, 188.

STEVEN, F. S., GRIFFIN, M. M., ITZHAKI, S. & AL-HABIB, A. (1981) The export of a trypsin inhibitor from Ehrlich ascites tumour cells which does not inhibit a trypsin-like enzyme on the surface of these cells. *Trans. Biochem. Soc.* (*In* press.)

TARIN, D. (1972) Morphological studies on the mechanism of carcinogenesis. In *Tissue Interactions in Carcinogenesis*. Ed. Tarin. London: Academic Press. p. 227.

TARIN, D. (1976) Cellular interactions in neoplasia. In *Fundamental Aspects of Metastasis*. Ed. Weiss. Amsterdam: North-Holland Publishing Co. p. 151.

VERLOES, R., ATASSI, C., DUMONT, P. & KANAREK, L. (1978) Tumour growth inhibition mediated by trypsin inhibitor or urokinase inhibitors. *Eur. J. Cancer*, **14**, 23.

WHUR, P., ROBSON, R. T. & PAYNE, N. E. (1973) Effects of protease inhibitor in the adhesion of Ehrlich ascites cells to host cells *in vivo*. *Br. J. Cancer*, **28**, 417.

WOOLLEY, D. E., TETLOW, L. C. & EVANSON, J. M. (1980) In *Collagenase in Normal and Pathological Connective Tissues*. Eds Woolley & Evanson. New York: John Wiley & Sons. p. 105.